# Comparative RNA-seq and functional assays identify L-arginine transporter SLC7A2 as a potential regulator of osteogenesis in maxillary sinus mucosal stem cells

**DOI:** 10.3389/fgene.2025.1701001

**Published:** 2025-10-28

**Authors:** Jing Ren, Ningbo Geng, Tianren Zhou, Shiting Mo, Chi Zhang, Wei Peng, Chunhui Liao, Songling Chen

**Affiliations:** ^1^ Department of Stomatology, The First Affiliated Hospital of Sun Yat-sen University, Guangzhou, Guangdong, China; ^2^ Department of Orthodontics, Guangzhou Women and Children’s Medical Center, Guangzhou, Guangdong, China

**Keywords:** comparative RNA-seq, MSMSCs, PMSCs, SLC7A2, amino acid metabolism, osteogenesis

## Abstract

**Background:**

The osteogenic differentiation of maxillary sinus mucosal stem cells (MSMSCs) plays a critical role in maxillary sinus floor elevation, yet its underlying regulatory mechanisms remain unclear. In addition, although both MSMSCs and palatal mesenchymal stem cells (PMSCs) may participate in bone regeneration, their osteogenic differentiation characteristics and molecular regulation differences have not been systematically analyzed. Therefore, the study aimed to characterize the transcriptional differences between MSMSCs and PMSCs during osteogenesis and identify the role of solute carrier family 7 member 2 (SLC7A2) in MSMSCs’ osteogenic differentiation.

**Methods:**

RNA sequencing (RNA-Seq) was conducted to compare gene expression profiles of MSMSCs and PMSCs at baseline (day 0) and 7, 14, and 21 days after osteogenic induction. Osteogenic differentiation was evaluated using Alkaline Phosphatase (ALP) staining, Alizarin Red S (ARS) staining, Western blotting, and quantitative polymerase chain reaction (qPCR) for the osteogenic markers osteocalcin (OCN), runt-related transcription factor 2 (RUNX2), and bone morphogenetic protein 2(BMP2). A lentiviral-based SLC7A2-silencing model was established in MSMSCs to silence SLC7A2 expression and thereby investigate its role in osteogenic differentiation.

**Results:**

MSMSCs and PMSCs exhibited similar osteogenic gene expression patterns, but their underlying regulatory mechanisms differed. Notably, amino acid metabolism-related pathways were significantly enriched in MSMSCs during osteogenesis. SLC7A2 was identified as one of the top 10 differentially expressed genes (DEGs) common to MSMSCs at baseline (day 0) and 7, 14, and 21 days after osteogenic induction (FDR <0.05 and |log_2_FC| ≥2.0). Functional experiments further demonstrated that SLC7A2 knockdown in MSMSCs resulted in a significant decrease in ARS staining and ALP activity, and significantly suppressed the expression of OCN and RUNX2 compared to the control shEV group (*P* < 0.05, n = 3).

**Conclusion:**

The osteogenic differentiation of MSMSCs is tightly associated with amino acid metabolism.Notably, SLC7A2—an L-arginine transporter—is a gene required for the efficient osteogenic differentiation of MSMSCs. This study provides novel evidence to advance our understanding of the molecular mechanisms underlying stem cell osteogenic differentiation during maxillary sinus floor lifting surgery,and further suggests that SLC7A2 may serve as a potential target to enhance the efficacy of MSMSCs-based bone regeneration.

## 1 Introduction

The mechanism of osteogenesis after maxillary sinus floor elevation has been a concern of scholars. The tissue structure and biological properties of maxillary sinus mucosa play an important role in this process. Some studies at home and abroad have shown that without bone grafting in maxillary sinus floor elevation, there can be new bone formation in the maxillary posterior region to support dental implants by elevating the maxillary sinus mucosa and maintaining its position (Berbéri et al., 2017; Lundgren et al., 2017). Our previous research has compared the morphology and molecular biology of the maxillary sinus mucosa and palatal mucoperiosteum, and preliminarily confirmed that the maxillary sinus mucosa is a type of mucoperiosteum rich in mesenchymal stem cells and has osteogenic ability (Ren et al., 2022). We have also successfully isolated, cultured, and identified maxillary sinus mesenchymal stem cells (MSMSCs) and palatine mesenchymal stem cells (PMSCs), and established osteogenic differentiation models of MSMSCs and PMSCs *in vitro*.

The formation of bone tissues is a highly complex process involving a series of gene expression changes. Moreover, mesenchymal stem cells (MSCs) from different tissues have different biological characteristics, and their positional memory functions and genetic expressions are different from each other (Kadkhoda et al., 2024; Naeem et al., 2022; Aasebø et al., 2021; Ghasemzadeh et al., 2018). Although both MSMSCs and PMSCs have osteogenic differentiation potential, the intensity and speed of osteogenesis differ at different time points after osteogenesis induction (Ren et al., 2022). The gene expressions underlying phenotypic changes have not been clarified, especially changes at the transcription level during osteogenic differentiation. MSMSCs and PMSCs originate from anatomically contiguous regions: the maxillary sinus and hard palate respectively. Anatomically, these sites reside in the upper and lower maxilla within the oral and maxillofacial complex. Extensive research on PMSCs has been performed globally, demonstrating their clinical utility in applications such as bone defect repair in cleft palate treatment and jaw reconstruction (Natsir Kalla et al., 2024; Leelan et al., 2023). While several clinical trials and animal studies have highlighted the osteogenic differentiation potential of maxillary sinus mucosa, however,whether MSMSCs exhibit comparable or superior efficacy to PMSCs in promoting maxillary bone regeneration remains undefined. Thus, investigating transcriptional and expression profiles during osteogenic differentiation of MSMSCs and PMSCs is critical to elucidate their osteogenic differentiation potential and gene expression patterns (Wang et al., 2022). These insights may expand the therapeutic scope of stem cell therapy in oral and maxillofacial surgery and orthopedics, offering novel clinical strategies for managing vertical bone deficiency in the maxillary posterior region.

It is known that osteogenic differentiation is a complex and orchestrated process, during which an array of cytokines, signaling pathways, and small molecules drive MSCs to differentiate into osteoblasts, which subsequently mature, secrete extracellular matrix, and mineralize bone (Basoli et al., 2021; Narcisi et al., 2021). Meanwhile, the intracellular metabolism of glucose, fatty acids, and amino acids undergoes dynamic reprogramming during MSC osteogenic differentiation. Emerging evidence indicates that amino acid metabolism, such as that of arginine, is critically associated with MSC osteogenesis (Chevalley et al., 1998; Tabbaa et al., 2021). Amino acids and their transporters not only serve as nutritional sources but also function as signaling molecules in cells. Certain transporters can initiate signal transduction cascades during amino acid substrate transport. In embryonic stem cells, amino acid metabolites regulate DNA and specific epigenetic histone modifications to maintain stem cell proliferation and pluripotency (Lon and Rebecca, 2019). These observations emphasize the necessity of maintaining optimal amino acid levels and transporter activity for sustaining diverse biological processes in MSCs, including osteogenic differentiation.

Solute carrier family 7 member 2 (SLC7A2), a key transporter for L-arginine uptake, is localized on chromosome 8 and spans 76,505 nucleotides with 12 exons. The SLC7A2 protein is membrane-bound, consisting of 658 amino acids with a molecular weight of 72 kDa. L-arginine(Arg), a semi-essential amino acid, serves as a precursor for the synthesis of proteins, polyamines, and nitric oxide (NO). Previous studies have demonstrated that NO enhances bone morphogenetic protein 2 (BMP-2)-induced osteogenesis via the Smad signaling pathway (Differ et al., 2019). Arginine metabolism also regulates mammalian target of rapamycin (mTOR) pathway, a critical pathway regulator of MSC differentiation. Notably, the mTOR signaling pathway modulates the activities of osteogenesis-related transcription factors such as Runt-related transcription factor 2 (RUNX2) and promotes the expression of osteogenic proteins like Osteocalcin (OCN). This enhances their nuclear localization and osteogenic gene transcription. Emerging evidence shows that SLC7A2 acts as a biomarker and an independent prognostic indicator for multiple cancers, including breast, colon, and liver cancer (Coburn et al., 2016; Coburn et al., 2019). Moreover, SLC7A2 may contribute to aberrant cell differentiation and proliferation in tumor microenvironments, which could potentially intersect with osteogenic regulatory networks. Additionally, SLC7A2 plays a pivotal role in skeletal muscle differentiation and represents a promising therapeutic target for sarcopenia (Huang et al., 2023; Chen et al., 2018).

At present, the specific mechanisms underlying the osteogenic potential of MSMSCs remain unclear. The role of amino acid metabolism and its transporters in the osteogenic differentiation of MSCs has been gradually revealed, but the function of amino acid metabolism in the osteogenic differentiation of MSMSCs has not been thoroughly studied. In this study, comparative RNA sequencing (RNA-seq) analysis was used to explore the differences in gene expression between MSMSCs and PMSCs to identify the molecular pathways regulating the different biological characteristics of the two types of stem cells. In addition, the genes that may play key roles in the process of osteogenic differentiation of MSMSCs were further identified by precise setting of time points. Finally, this study for the first time identified the key role of SLC7A2 in osteogenic differentiation of MSMSCs and revealed the molecular mechanism by which SLC7A2 promotes osteogenic differentiation by regulating L-arginine metabolism and mTOR signaling pathway. These findings provide a theoretical basis for improving the osteogenic differentiation capacity of MSMSCs and offer new strategies for promoting site-specific osteogenesis after maxillary sinus elevation.

## 2 Materials and methods

### 2.1 Isolation, culture and identification of MSMSCs and PMSCs

In the present study, all specimens were obtained from the Department of Stomatology, First Affiliated Hospital of Sun Yat-sen University. MSMSCs were isolated from maxillary sinus mucosal tissue of patients undergoing Le Fort I Osteotomy (maxillary orthognathic surgeries), and PMSCs from hard palate mucoperiosteum of patients undergoing palatal surgeries. Inclusion/exclusion criteria and methodologies are detailed in our previous study (Ren et al., 2022). This study was approved by the Medical Ethics Committee of the First Affiliated Hospital of Sun Yat-sen University (Ethics Approval No. 2021212).

Fresh Schneiderian membranes and palatal periosteum were placed in MEM-α medium (Gibco, United States), washed with DPBS (Genview, China), and digested with 150 U/mL collagenase type II (Sigma, United States) in 5 mL DMEM/F12 (1:1) at 37 °C for 45 min. The digested suspension was filtered through a 70-μm strainer, washed with MEM-α (supplemented with 10% FBS and 1% Antibiotic-Antimycotic; Gibco, United States), and centrifuged at 300 *g* for 5 min. Cells were resuspended in MEM-α (10% FBS, 1% Antibiotic-Antimycotic, 2 mM glutamine) and seeded at 1 × 10^5^ cells/well in six-well plates. Cultures were maintained at 37 °C with 5% CO_2_, with medium changes every 2 days and passaging every 5–7 days. Finally, MSMSCs and PMSCs were obtained as primary cells (Primary cell, Pr).

### 2.2 Osteogenic differentiation, alkaline phosphatase (ALP), and Alizarin Red S staining (ARS) of MSMSCs and PMSCs

The second-passage (P2) MSMSCs and PMSCs were seeded in 6-well plates at 1 × 10^5^ cells/well and cultured at 37 °C with 5% CO_2_. When reaching 80%–90% confluence, osteogenic medium (StemCell Technologies, Canada), prepared according to themanufacturer’s instructions, was added to each well. Cells were cultured in osteogenic medium for 21 days, with medium changed every 3 days.

Osteogenic differentiation was evaluated by Alkaline Phosphatase (ALP) and Alizarin Red S (ARS) staining at 21 days post-induction. Residual medium was removed, and wells were washed with DPBS. Cells were fixed with 4% paraformaldehyde (Beyotime, China) for 10 min at room temperature, then rinsed 2–3 times with ddH2O to remove residual fixative. For staining: 1 mL ARS (Cyagen Biosciences, United States) was added per well and incubated in the dark for 15 min at room temperature; alternatively, 400 μL ALP staining solution (Gibco, United States) was added and incubated for 15 min at room temperature. After staining, unbound dye was removed by washing three times with ddH2O (3–5 min per wash). Results were observed and recorded under an inverted microscope (Leica, Germany) at appropriate magnifications.

### 2.3 Immunofluorescent staining of MSMSCs and PMSCs

MSMSCs and PMSCs at day 0 (pre-osteogenic induction) and day 21 (post-osteogenic induction) were digested with 0.25% trypsin to prepare single-cell suspensions. Suspensions (100 μL PBS per slide) were centrifuged onto glass slides (20,000 cells/slide) using a cytocentrifuge, air-dried at room temperature, and cell areas delineated with a PAP pen. Immunofluorescence staining (25 °C) proceeded as: fixed in 4% paraformaldehyde for 10 min, permeabilized with 0.25% Triton X-100 for 10 min, and washed with PBS three times; blocked with normal goat serum for 45 min; incubated with primary antibody against Osteocalcin (OCN; Affinity, 1:100) overnight at 4 °C, then PBS-washed three times; labeled with Alexa Fluor^®^ 594-conjugated goat anti-rabbit IgG (H + L; Thermo Fisher) for 60 min in the dark, followed by three PBS washes; counterstained with DAPI for 5 min and final PBS washes. Slides were coverslipped with antifade mounting medium, and fluorescent images were captured using a fluorescence microscope (Olympus BX53, Japan) to analyze the expression levels of cell surface markers in each sample.

### 2.4 RNA extraction and sequencing

RNA-seq was performed following standard protocols (RNA extraction, library preparation, quality assessment). MSMSCs and PMSCs were selected as experimental subjects, with each cell type assigned to independent groups (Group M for MSMSCs; Group B for PMSCs). Cells at passage 2 (P2), seeded at a density of 1 × 105 cells/well, were cultured until they reached approximately 80%–90% confluence. Total RNA was isolated from 24 samples using the TRIzol kit (Thermo Fisher, United States). The isolation process was carried out at four specific time points: before osteogenic induction (day 0), and 7, 14, and 21 days after osteogenic induction. After isolation, RNA samples were stored at −80 °C until all samples were collected for library construction and sequencing.

RNA quality was assessed using Agilent Bioanalyzer (Agilent 2100, United States). It was checked using RNase-free agarose gel-electrophoresis, and RNA quality was determined by RNA integrity (RIN). The eukaryotic mRNA with polyA was enriched using magnetic Oligo (dT) beads, then fragmented in buffer. First-strand cDNA was synthesized with random oligonucleotide primers in an M-MuLV reverse transcriptase system (using fragmented mRNA). Second-strand cDNA was synthesized from deoxynucleotides after RNA degradation with ribonuclease. Next, the purified double-stranded cDNA was end-repaired, and A base was added and ligated to Illumina sequencing adapters. The ligation reaction was purified with the AMPure XP Beads (1.0X).

### 2.5 RNA sequencing analysis

In the RNA-seq data analysis of this study, the cDNA library was sequenced using Illumina Novaseq 6000 by Gene Denovo Biotechnology Co. (Guangzhou, China). The average sequencing depth for each sample was 40 million reads. Reads were further filtered by fastp (v.0.18.0) and paired-end clean reads were mapped to the reference genome using HISAT2 (v.2.1.0) and other parameters set as a default. The differentially expressed genes (DEGs) were analyzed by using DESeq2 software. The genes with the parameter of false discovery rate (FDR) <0.05 and absolute log_2_ (fold change) ≥2 were considered differentially expressed genes.

All statistical analyses were conducted using the R programming language (version 3.6.2, http://www.R-project.org). Gene expression levels were quantified as read counts and fragments per kilobase of exon per million mapped reads (FPKM). To further explore the functional implications of the DEGs, the “clusterProfiler” package was used, which enabled Gene Ontology (GO) and Kyoto Encyclopedia of Genes and Genomes (KEGG) pathway enrichment analysis of these DEGs. This provided insights into the biological processes, molecular functions, cellular components, and pathways associated with the observed gene expression changes.

### 2.6 Construction of lentivirus and transfection

Short hairpin RNA targeting solute carrier family 7 member 2 (shSLC7A2) is a short hairpin RNA (shRNA) expression vector specifically designed to target the SLC7A2 gene. It was constructed using the PLKO.1-puro vector backbone (Hanbio, Shanghai, China) to mediate SLC7A2 knockdown via RNA interference (RNAi). The empty PLKO.1-puro vector served as the shRNA empty vector (shEV).

PLKO.1 vector (8,415 bp) was propagated in LB medium (Sangon Biotech, China) supplemented with 50 μg/mL ampicillin (1:1000 bacterial inoculum), shaken at 200 rpm for 12–16 h at 37 °C. Plasmid DNA was extracted using EndoFree Plasmid Midi Kit (CoWin Biotech#CW2105S, China) and verified by gel electrophoresis for integrity and concentration. The vector was double-digested and dephosphorylated overnight at 37 °C, heat-inactivated at 65 °C for 20 min, mixed with 6X DNA loading buffer, and stored at 4 °C. After confirming the target fragment by 1% gel electrophoresis, the 8.5-kb fragment was recovered using Gel Extraction Kit (Omega#D2500-01, United States). Complementary DNA oligos were annealed via thermal cycler: 37 °C for 30 min, 95 °C for 6 min, then ramped down to 25 °C at 0.1 °C/s. The annealed oligos were diluted 2.5-fold to 50 μL and stored at 4 °C. Ligation was performed by incubating annealed insert with linearized vector at 18 °C for 6 h, followed by 30 min at room temperature and storage at −20 °C.

The DNA was chemically transformed using TransStbl3 Chemically Competent Cells (Shanghai Maokang Biotechnology Co., China). 50 μL thawed competent cells were placed on ice, gently mixed with target DNA, and incubated on ice for 20–30 min. After 45-s heat shock at 42 °C, cells were transferred immediately to ice for 2 min 500 μL sterile antibiotic-free LB medium was added, and cells were incubated at 37 °C with shaking (200 rpm) for 1 h for recovery. 100 μL and 200 μL of culture were plated onto antibiotic-supplemented LB agar plates, spread evenly, and plates were inverted and incubated overnight at 37 °C (∼10 colonies observed per plate). Three single colonies per plate were selected for Sanger sequencing (BGI) using universal U6 primers.

In this experiment, a total of 4 shSLC7A2 lentivirus vectors were successfully constructed, and the target sequences used in this study are provided in Table 1.

**TABLE 1:** Target sequences of shSLC7A2 lentiviral vectors.

Number	Sense strand sequence (5′→3′)	Antisense strand sequence (5′→3′)
shSLC7A2#1	CCG​GGC​TGG​GTT​TGT​GAA​AGG​AAA​TCT​CGA​GAT​TTC​CTT​TCA​CAA​ACC​CAG​CTT​TTT​G	AAT​TCA​AAA​AGC​TGG​GTT​TGT​GAA​AGG​AAA​TCT​CGA​GAT​TTC​CTT​TCA​CAA​ACC​CAG​C
shSLC7A2#2	CCG​GCG​TGA​GCT​TTC​TGG​TAG​GAT​TCT​CGA​GAA​TCC​TAC​CAG​AAA​GCT​CAC​GTT​TTT​G	AAT​TCA​AAA​ACG​TGA​GCT​TTC​TGG​TAG​GAT​TCT​CGA​GAA​TCC​TAC​CAG​AAA​GCT​CAC​G
shSLC7A2#3	CCG​GCG​TAT​GTG​ATA​GGT​ACA​TCA​ACT​CGA​GTT​GAT​GTA​CCT​ATC​ACA​TAC​GTT​TTT​G	AAT​TCA​AAA​ACG​TAT​GTG​ATA​GGT​ACA​TCA​ACT​CGA​GTT​GAT​GTA​CCT​ATC​ACA​TAC​G
shSLC7A2#4	CCG​GGC​CCA​AAT​GTT​CTC​CTG​AGA​ACT​CGA​GTT​CTC​AGG​AGA​ACA​TTT​GGG​CTT​TTT​G	AAT​TCA​AAA​AGC​CCA​AAT​GTT​CTC​CTG​AGA​ACT​CGA​GTT​CTC​AGG​AGA​ACA​TTT​GGG​C

Lentiviral transfection of MSMSCs and sample collection: shSLC7A2 and control shEV groups were transfected into MSMSCs at passage 3 (P3). After digestion and counting, 1 × 10^5^ cells/well were seeded in 12-well plates (one unseeded well as blank control). The final concentration of the transfection-promoting reagent polybrene (Hanbio Biotechnology, China) was 10 μg/mL, and the virus solution to culture medium ratio was at least 1:3 to avoid nutrient dilution. 24 h after transfection, medium was replaced with 1 μg/mL Puromycin Dihydrochloride (Beyotime, China) for 2-day selection.

Following lentiviral packaging and transfection of MSMSCs, cell morphology was observed via mCherry fluorescence using an inverted microscope (Leica, Germany). Cell viability was assessed using Calcein/Pl Live/Dead Cytotoxicity Assay Kit (Beyotime, #C2015S, China) and Fixable Viability Dye eFluor™ 780 (Thermo Fisher Scientific, #65-0865-14, United States) according to the manufacturer’s instructions. Flow cytometry analysis was conducted using BD flow cytometer (BD Biosciences,United States). For shSLC7A2 and shEV groups, digested cells were transferred to 12 × 75 mm tubes, washed twice with azide/protein-free PBS, and resuspended at 1–10 × 10^6^ cells/mL in azide/serum-free PBS. 1 μL FVD per 1 mL suspension was added, vortexed immediately, and stained at 2 °C–8 °C in the dark for 30 min. Cells were washed 1–2 times with flow cytometry staining buffer. Flow cytometry was performed with specific settings: FVD780 (excited at 633 nm, detected with 780/60 filter); Calcein (excited at 488 nm, detected with 525/40 filter).

shSLC7A2 and shEV groups were cultured under standard conditions for 72 h transfection efficiency of shSLC7A2 was confirmed using Western blot (WB) and quantitative PCR (qPCR) to ensure successful knockdown of SLC7A2. The expressions of SLC7A2 (ABclonal) were detected. 72 h after transfection, cells were digested into single-cell suspensions, centrifuged to collect, washed twice with PBS, and used for RNA extraction with TRIzol kit (Thermo Fisher, United States). Reverse transcription was then performed using PrimeScript RT kit (Takara, Japan). During the experiment, the fluorescence expression of the transfected cells and overall growth status were continuously monitored, with regular digital images taken for analysis.

### 2.7 Osteogenic differentiation staining of MSMSCs transfected with shSLC7A2

shSLC7A2 and shEV groups were seeded in six-well plates at 1 × 10^5^ cells/well. When cells reached ∼80–90% confluence, medium was replaced with osteogenic induction medium (StemCell Technologies, Canada) for differentiation. After 21 days of induction, ALP and ARS staining were performed to assess mineralized calcium content: Cells were fixed with 4% paraformaldehyde at 25 °C for 10 min, rinsed with DPBS, then stained with 1 mL/well ARS solution (Cyagen) or 400 μL/well ALP dye (Solarbio) for 15 min, and rinsed three times with deionized water. Detailed procedures refer to Section 2.2.

### 2.8 Western blot (WB) and quantitative PCR (qPCR)

To further assess osteogenic differentiation potential, shSLC7A2 and shEV groups were cultured in 6-well plates with 2 mL osteogenic induction kit medium (StemCell Technologies, Canada) per well. At 0, 7, 14, and 21 days post-induction, total proteins were extracted: cells were lysed with RIPA buffer, lysates centrifuged at 16,000 g for 10 min at 4 °C, and supernatant proteins collected. Protein concentration was quantified via BCA method, adjusted with 5× loading buffer, and denatured at 99 °C for 5 min. The samples were then subjected to SDS-PAGE electrophoresis on 5% polyacrylamide gels, with 15 μg of protein loaded per well. Proteins were wet-transferred to PVDF membranes, which were blocked with 5% skim milk at room temperature for 2 h. The membranes were incubated overnight at 4 °C with polyclonal antibodies against human RUNX2 (Affinity Biosciences, Catalog No.:AF7879, 1:1000 dilution), BMP2 (Affinity Biosciences, Catalog No.: AF5163, 1:1000 dilution), OCN (Affinity Biosciences, Catalog No.: DF12303, 1:1000 dilution), and SLC7A2 (ABclonal, Catalog No.: A14574, 1:500 dilution), followed by incubation with secondary antibody Goat Anti-Rabbit IgG (H + L) HRP (Affinity Biosciences, Catalog No.: S0001, 1:2000 dilution) for 1 h at room temperature. Protein bands were visualized with enhanced chemiluminescence, imaged using Alpha Innotech system, and quantified via relative gray value analysis.

At 3, 7, 14, and 21 days after osteogenic differentiation, total cellular RNA was extracted using TRIzol kit (Thermo Fisher, United States) according to manufacturer’s protocol: Cells were washed twice with ice-cold PBS, lysed in 1 mL TRIzol, and homogenized by pipetting. Samples were incubated at room temperature for 15 min to dissociate nucleoprotein complexes. Following centrifugation at 12,000 × g for 15 min at 4 °C, the aqueous phase was transferred to an RNase-free tube. RNA was precipitated with 500 μL isopropanol, mixed by inversion, incubated for 15 min at room temperature, and pelleted by centrifugation at 12,000 × g for 10 min at 4 °C. The pellet was washed with 1 mL 75% ethanol, air-dried, and resuspended in DEPC-treated water. RNA concentration and purity were assessed using a NanoDrop 2000 spectrophotometer (Thermo Fisher #Nanodrop 2000; United States). Then cDNA was synthesized by reverse transcription of total RNA following the detailed instructions of the PrimeScript RT kit (Takara, Japan). The reaction was performed in a 20 μL volume, containing 1 μg of mRNA and 5 μL of SuperMix, with the volume adjusted to 20 μL using DEPC water. The mixture was incubated at 42 °C for 15 min, then at 5 °C for 5 s, immediately placed on ice, and stored at −20 °C for later use. qPCR was performed in a 96-well plate using BIO-Rad IQ5 instrument (United States) with cycling conditions: initial denaturation at 94 °C for 30 s, followed by 40 cycles of 94 °C for 5 s and 60 °C for 30 s (annealing/extension). Melting curve analysis confirmed PCR product specificity.

The results of qPCR were averaged from three technical replicates and analyzed using the Bio-Rad qPCR system (Bio-Rad IQ5, United States). Glyceraldehyde 3-phosphate dehydrogenase (GAPDH) was selected as the housekeeping gene for qPCR normalization. The relative expression levels of target genes in each group were quantified using the 2^−ΔΔCt method with GAPDH as the reference gene. The expressions of SLC7A2 (ABclonal), RUNX2 (Affinity), BMP2 (Affinity), and OCN (Affinity) were detected. All primers were designed using Primer-BLAST (NCBI) to ensure specificity and synthesized by Sangon Biotech (Shanghai, China). Amplification efficiency for each primer pair was validated via standard curve analysis. The primer sequences used in this study are provided in Table 2.

**TABLE 2:** Primer sequences for qPCR analysis.

Gene name	Forward primer (5′→3′)	Reverse primer (5′→3′)
SLC7A2	GAC​CTT​TGC​CCG​ATG​TCT​GAT	AGC​AGC​GGC​ATA​ATT​TGG​TGT
RUNX2	GAATGCCTCTGCTGTTAT	TTGTGAAGACGGTTATGG
BMP2	ACC​CGC​TGT​CTT​CTA​GCG​T	TTT​CAG​GCC​GAA​CAT​GCT​GAG
OCN	AGGGCAGCGAGGTAGTGA	CCTGAAAGCCGATGTGGT

### 2.9 Statistical analysis

Data are presented as mean ± standard deviation (SD) from at least three independentbiological replicates. Statistical analyses were performed using SPSS software version 20.0(SPSS, Inc., United States). A two-way mixed-design analysis of variance (ANOVA) was used to assess the interactions between experimental factors, followed by Tukey’s *post hoc* test for pairwise comparisons. Comparisons between multiple groups were using one-way ANOVA. *P* < 0.05 was considered to indicate a statistically significant difference.

## 3 Results

### 3.1 Osteogenic differentiation and immunofluorescent staining of MSMSCs and PMSCs

**FIGURE 1:**
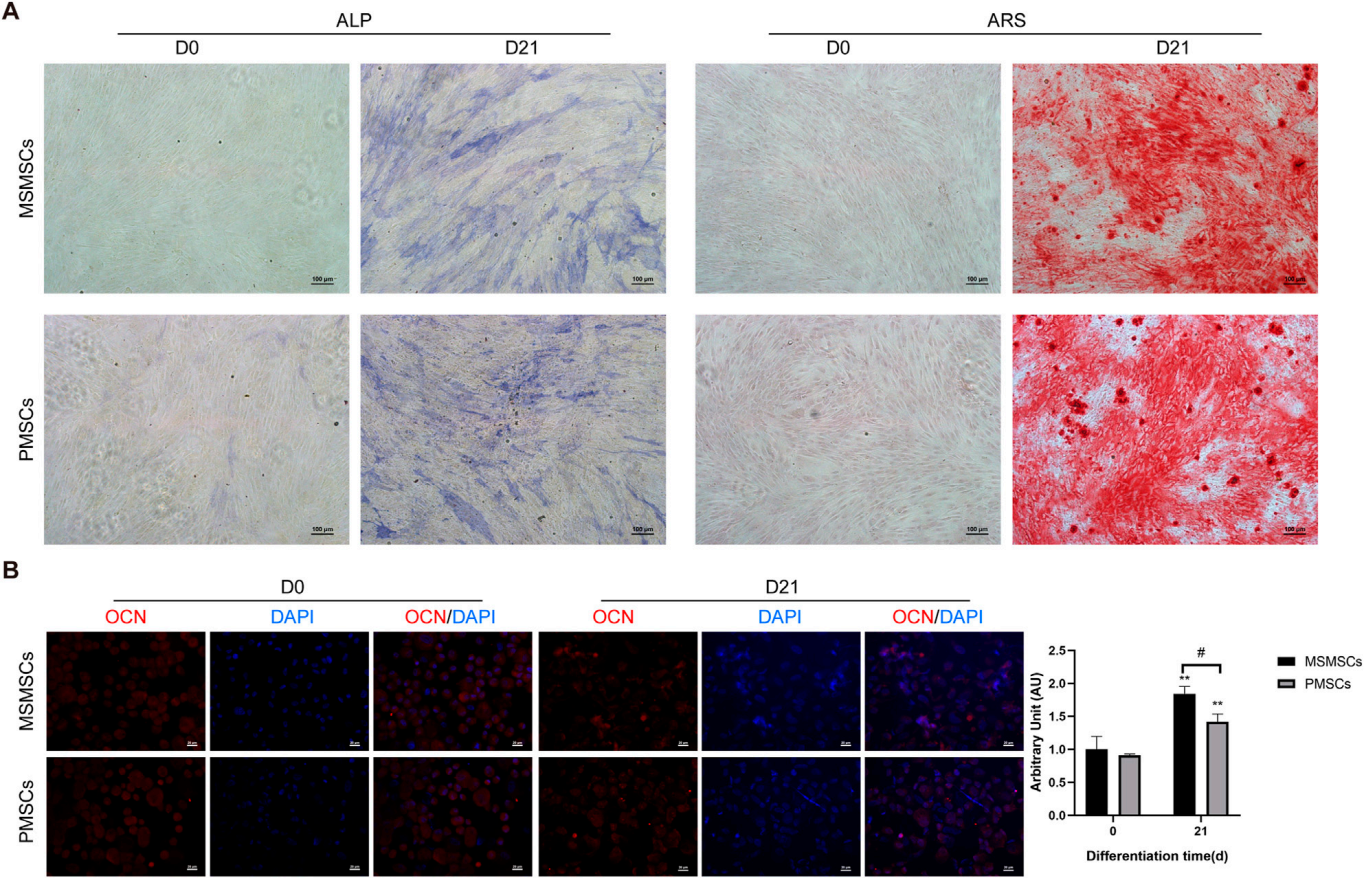
Osteogenic differentiation and immunofluorescent staining of MSMSCs and PMSCs. **(A)** ALP and ARS staining results of MSMSCs and PMSCs before (day 0) and 21 days after osteogenic induction. Scale bar = 100 μm. **(B)** Immunofluorescence staining for OCN in MSMSCs and PMSCs before and 21 day:Red: OCN-stained cells; Blue: DAPI-stained nuclei. Fluorescence intensity was semi-quantified by mean fluorescence intensity (MFI). ** indicate P < 0.01 when comparing D21 with D0 within the same group (n = 3, mean ± SD). # indicate P < 0.05 for intergroup comparison between MSMSCs and PMSCs at D21. Scale bar = 20 μm.Legend: ALP: Alkaline Phosphatase; ARS: Alizarin Red S; OCN: Osteocalcin; DAPI: 4′,6-diamidino-2-phenylindole.

Following osteogenic induction, MSMSCs and PMSCs underwent robust osteogenic differentiation, as demonstrated by ALP activity and ARS staining. By day 21, both cell types exhibited prominent calcium salt nodules with intense positive staining, indicative of mature osteoblast phenotypes and comparable osteogenic potential between MSMSCs and PMSCs in vitro (Figure 1A).

Immunofluorescent staining for osteogenic marker OCN revealed robust expression in both cell types after 21 days of osteogenic stimulation. Nuclei were counterstained with 4′,6-diamidino-2-phenylindole (DAPI) to visualize cellular density. Semi-quantitative analysis via mean fluorescence intensity (MFI) showed no significant difference in OCN/DAPI fluorescence expression levels between the two groups at day 0 (before induction). After 21 days of osteogenic induction, both MSMSCs and PMSCs exhibited significantly higher MFI values compared to day 0 (P < 0.01), with MSMSCs demonstrating significantly higher expression than PMSCs (P < 0.05) (Figure 1B).

Collectively, these results indicate that both MSMSCs and PMSCs possess osteogenic differentiation potential under standardized induction conditions, suggesting their utility for bone tissue engineering applications.

### 3.2 Sample testing, quality control and sample relationship analysis

**FIGURE 2:**
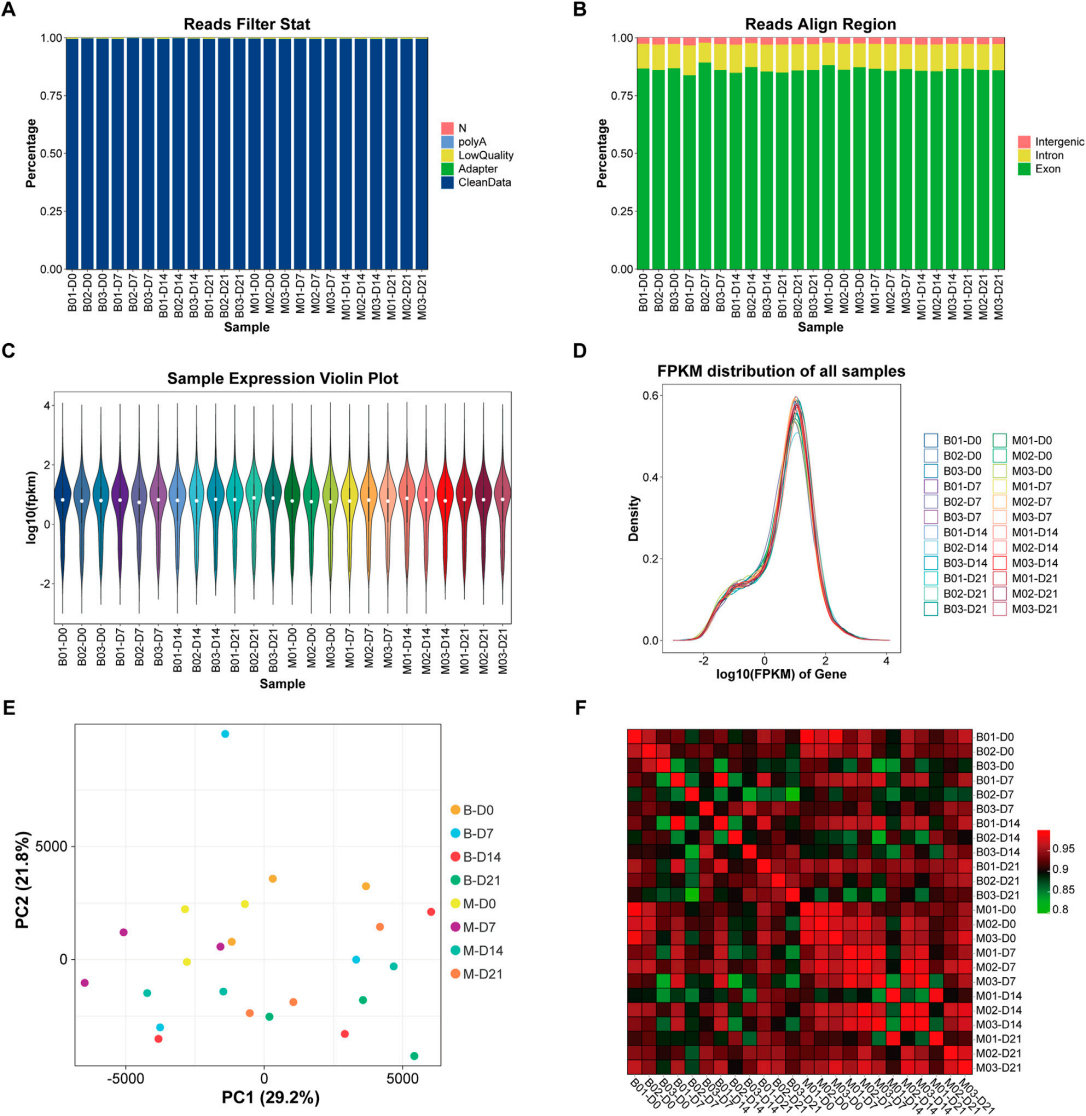
Sample detection, quality control, and sample relation analysis. **(A)** The scheme of data preprocessing. **(B)** The distribution of sequencing reads in the genome region of the 24 samples. **(C)** Violin plot of gene expression. ①The white dots represent the median Q2; ②The length represents the degree of dispersion and symmetry of non-abnormal data, longer lengths indicate data dispersion, while shorter lengths indicate concentration; The black lines running up and down the violin chart represent the interval from the minimum non-outlier to the maximum non-outlier, and the upper and lower ends of the lines represent the upper and lower limits, respectively. **(D)** Gene expression abundance distribution: the horizontal axis represents log10 (FPKM); the vertical axis represents the number of genes expressed. Each color represents a sample. **(E)** PCA analysis diagram: the horizontal axis represents the first principal component; the vertical axis represents the second principal component. **(F)** Correlation heat map: the horizontal and vertical axis represent 24 samples respectively, and the color depth indicates the magnitude of the Pearson’s correlation coefficient of the two samples. Legend: FPKM = fragments per kilobase of exon per million mapped reads; PCA = principal component analysis.

Twenty-four samples of MSMSCs and PMSCs at 0 (before), 7, 14, and 21 days after osteogenic differentiation were defined as Class A, and the RIN value of each sample was >9, indicating qualification of the RNA quality. There were 20,323 genes in the human genome in the 24 samples, and the unmapped reads were reduced to <5% by filtering low-quality data, and >95% clean reads were obtained (Lon and Rebecca, 2019). The scheme of data preprocessing is shown in (Figure 2A). The distribution of sequenced reads in the genome region of the 24 samples is shown in (Figure 2B). The average proportion of exons, introns, and intergenic regions mapped to the human genom was 86.25%, 11.03%, and 2.72%, respectively. Based on the FPKM values of each gene, the expression distribution of genes in different samples was visualized by gene expression profiles shown by expression profile (Figures 2C,D).

Based on principal component analysis (PCA) (Jolliffe and Cadima, 2016), 3 samples in M-D0 group were in the upper left quadrant, and 3 samples in B-D0 group were in the upper right quadrant. As the time of osteogenesis induction increased, shifts in principal components were observed and the samples M-D7, 14, and 21, and B-D7, 14, and 21 gradually moved to the lower right quadrant. The expression of any two of the 24 samples was used to calculate the Pearson correlation coefficient between every two samples, and the results were displayed as a heat map. The color intensity in the heat map represents the magnitude of the correlation, with darker colors representing higher correlations (Figures 2E,F).

### 3.3 Comparison of stem cell characteristics between MSMSCs and PMSCs

**FIGURE 3:**
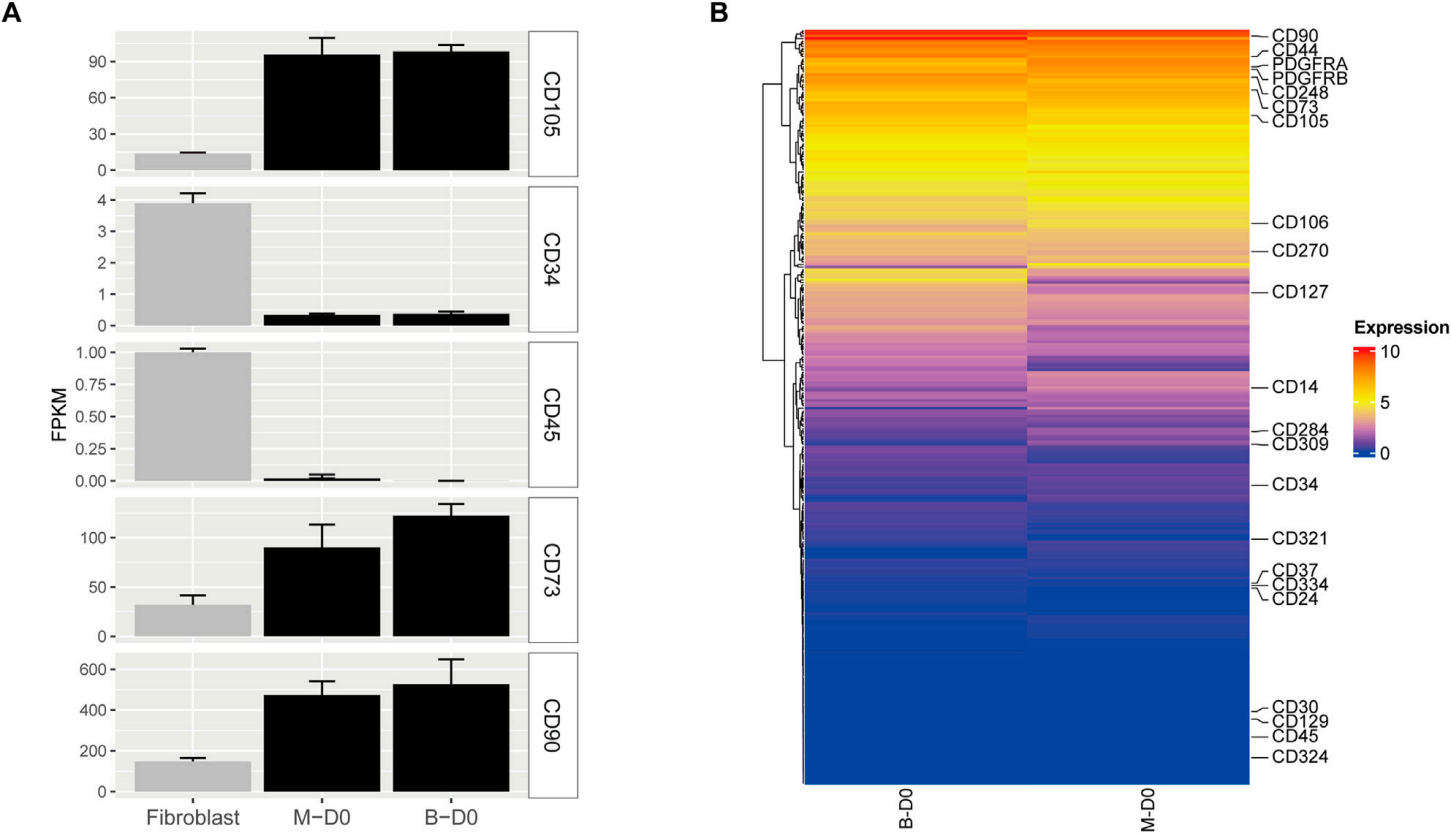
Gene expression profiles of CD markers in MSMSCs and PMSCs. **(A)** Expression levels of CD105, CD73, CD90, CD34, and CD45 in each cell type. RNA-seq data from human fibroblasts (GSE72229) were used as a control. **(B)** Heat maps of CD marker genes in MSMSCs and PMSCs; the vertical axis represents FPKM expression levels. Red indicates upregulated genes, and blue indicates non-differentially expressed genes (non-DEGs) between groups. Legend: “B-D0” = PMSCs at day 0; “M-D0” = MSMSCs at day 0; CD = Cluster of Differentiation; FPKM = fragments per kilobase of exon per million mapped reads; GSE = Gene Expression Omnibus Series.

To further verify that MSMSCs and PMSCs express stem cell-specific Cluster of Differentiation (CD) markers, human fibroblasts (data from GSE72229) were used as a control. The control fibroblast dataset was retrieved from the Gene Expression Omnibus Series (GSE) repository, with accession number GSE72229. The gene expression profiles of stem cell-specific surface markers were analyzed, including MSC-positive markers CD105 (ENG), CD73 (NT5E), CD90 (THY1), and negative markers CD34 and CD45 (PTPRC). These markers were analyzed in MSMSCs and PMSCs before osteogenic differentiation. RNA-seq expression was profiled in control fibroblasts using three biological replicates. The expression levels of CD105, CD73, and CD90 in both MSMSCs and PMSCs were higher than those in fibroblasts, while the levels of CD34 and CD45 were lower than those in fibroblasts (Figure 3A).

Heat maps revealed similar expression patterns of CD markers between MSMSCs and PMSC showed that the expression patterns were similar. CD90 had the highest expression (FPKM >400), and CD73 and CD105 were also highly expressed (FPKM >90). The expressions of CD14, CD34, and CD45 were low (FPKM <2.0), and CD182, CD69, and CD150 were not expressed (FPKM = 0) (Figure 3B).

### 3.4 Analysis of the differences between MSMSCs and PMSCs

**FIGURE 4:**
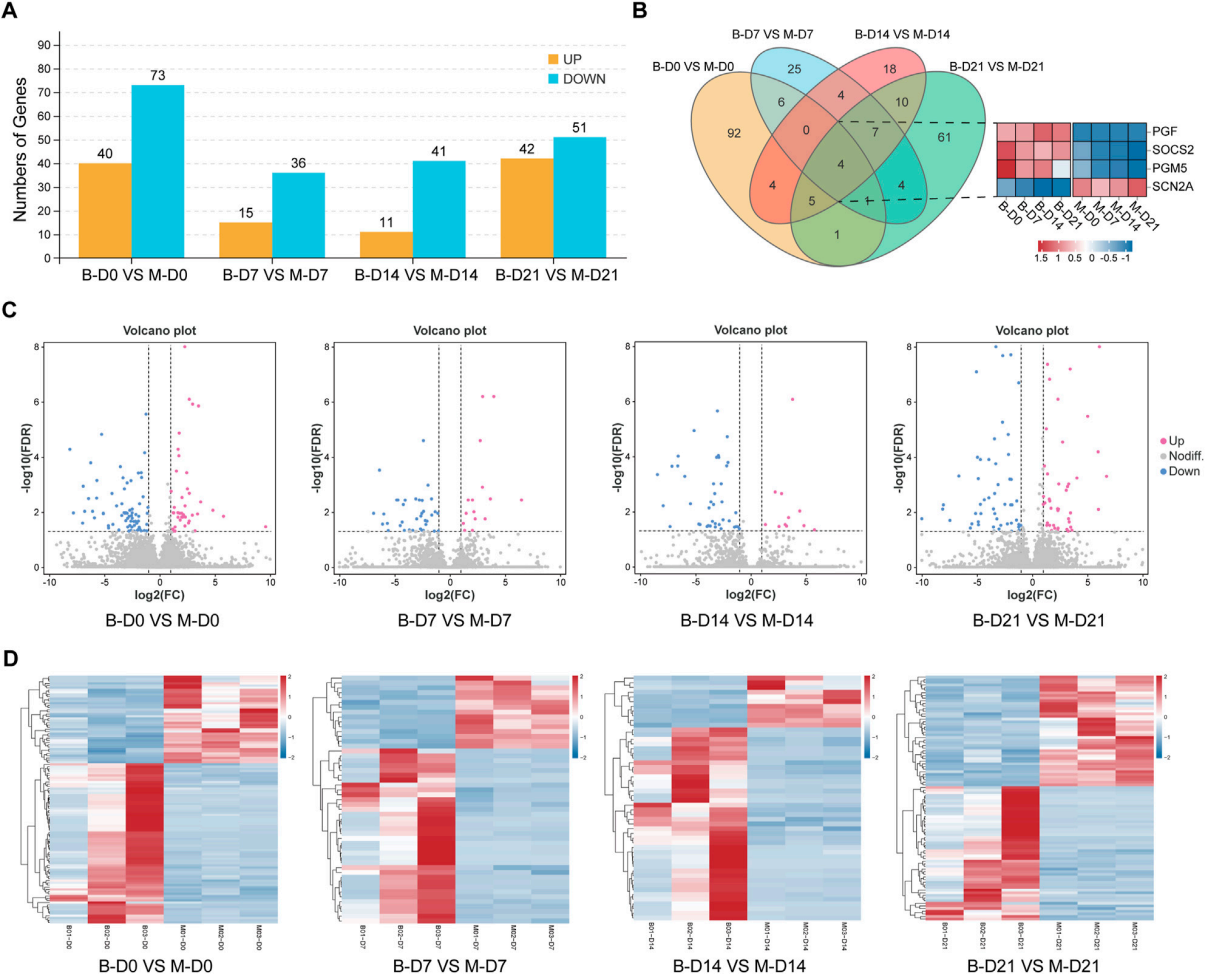
Analysis of the differences between MSMSCs and PMSCs. **(A)** Numbers of DEGs between PMSCs and MSMSCs at different time points. Yellow represents upregulated genes, and blue represents downregulated genes. **(B)** Venn diagram of DEGs comparing B-D0 vs. M-D0, B-D7 vs. M-D7, B-D14 vs. M-D14, B-D21 vs. M-D21. The number of overlapping regions represents the co-DEGs (Red represents upregulated genes, blue represents downregulated genes, and white represents non-DEGs, respectively). **(C)** Volcano plot showing all profiled genes. Red represents upregulated genes, blue represents downregulated genes, and gray represents non-DEGs, respectively. Statistical analysis detected DEGs between two samples (The judging criteria: FDR <0.05 and a more than two-fold change in FPKM). **(D)** Heat map showing the clustering of expression between PMSCs and MSMSCs at different time points. Red represents higher expression, blue represents lower expression, and white represents no change. Legend: “B-D0 vs. M-D0” = “PMSCs at day 0” versus “MSMSCs at day 0”; “B-D7 vs. M-D7” = “PMSCs at day 7” versus “MSMSCs at day 7”; “B-D14 vs. M-D14” = “PMSCs at day 14” versus “MSMSCs at day 14”; “B-D21 vs. M-D21” = “PMSCs at day 21” versus “MSMSCs at day 21”; DEGs = differentially expressed genes.

Based on the criteria of |log_2_FC| >2 and FDR <0.05, the DEGs in PMSCs and MSMSCs at different time points were screened using DESeq2 software (Love et al., 2014) (Figure 4A). Further comparison showed that MSMSCs and PMSCs had four common DEGs at 7, 14, and 21 days after osteogenesis induction, including three upregulated genes, PGF (vascular endothelial growth factor), SOCS2 (cytokine signaling inhibitor), PGM5 (phosphoglucomutase), and one downregulated gene, SCN2A (sodium channel subunit), linked to neuronal development but showing conserved repression in osteogenesis. These four genes all had the same trend of change at different detection time points in both cells and may act as conserved regulators of osteogenic divergence between MSMSCs and PMSCs (Figure 4B).

In order to determine the difference of transcript profiles between the two stem cells in the process of osteogenic differentiation, we arranged the different gene expressions at different times between the 2 groups as a volcano plot, respectively (Figure 4C). The expression patterns of the DEGs of the 24 samples were hierarchically clustered, and the clustering results were presented as a heat map. Before osteogenesis induction, and 7, 14, and 21 days after osteogenesis induction, the samples in MSMSCs and PMSCs groups using unsupervised clustering were consistent with those in the actual groups, indicating that there was less difference in gene expression between the two groups, and the experimental results exhibit good repeatability (Figure 4D).

### 3.5 Comparative GO and KEGG enrichment analysis of MSMSCs and PMSCs during osteogenic differentiation over time

**FIGURE 5:**
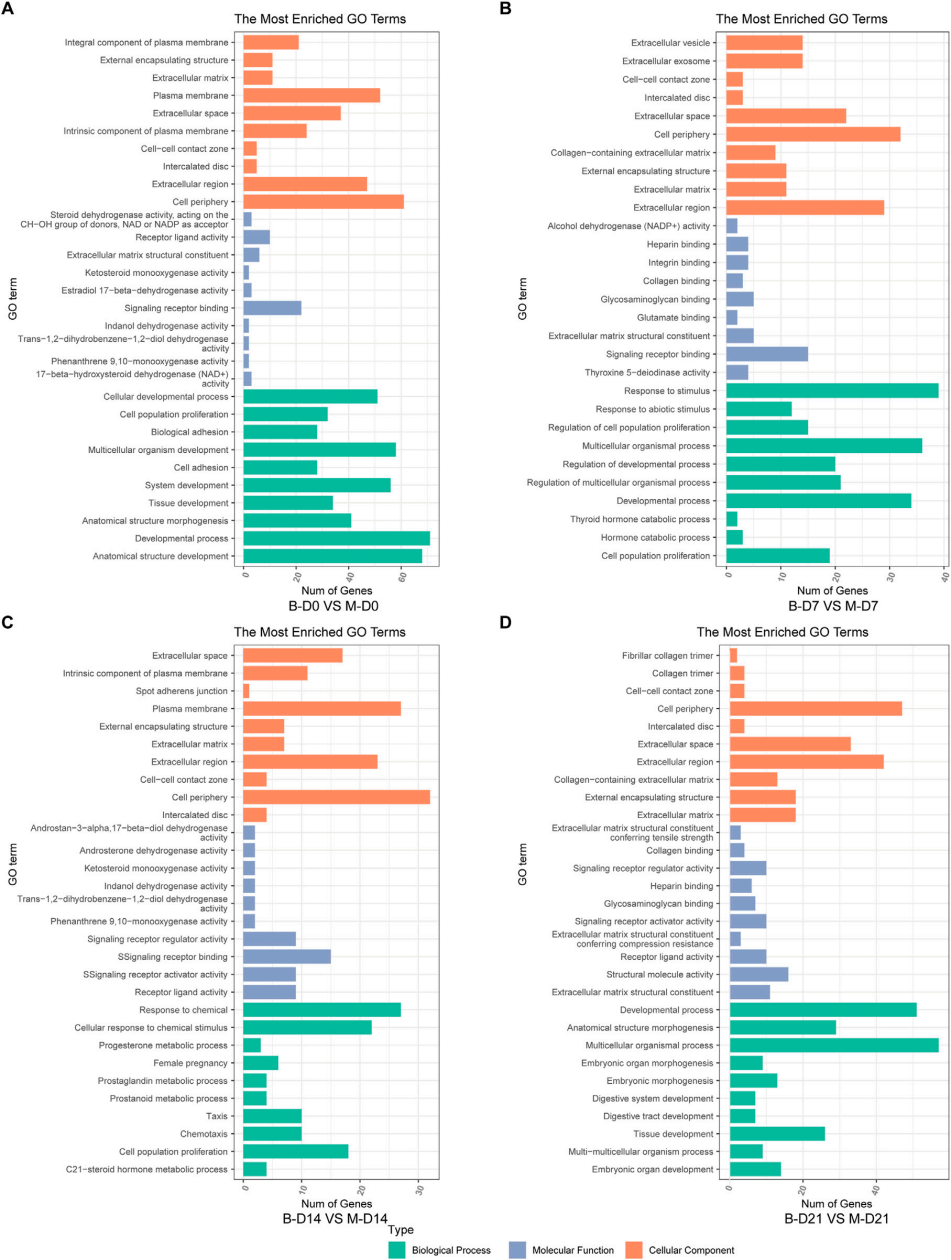
GO enrichment analysis of DEGs between MSMSCs and PMSCs. **(A)** Before osteogenic induction. **(B)** At 7 days after osteogenic induction. **(C)** At 14 days after osteogenic induction. **(D)** At 21 days after osteogenic induction. The top 10 most significantly enriched GO pathways at each time point were selected across three functional categories based on FDR <0.05: green denotes biological process, orange denotes cellular component, and blue denotes molecular function. Legend: “B-D0 vs. M-D0” = “PMSCs at day 0” versus “MSMSCs at day 0”; “B-D7 vs. M-D7” = “PMSCs at day 7” versus “MSMSCs at day 7”; “B-D14 vs. M-D14” = “PMSCs at day 14” versus “MSMSCs at day 14”; “B-D21 vs. M-D21” = “PMSCs at day 21” versus “MSMSCs at day 21”; GO = Gene Ontology.

**FIGURE 6:**
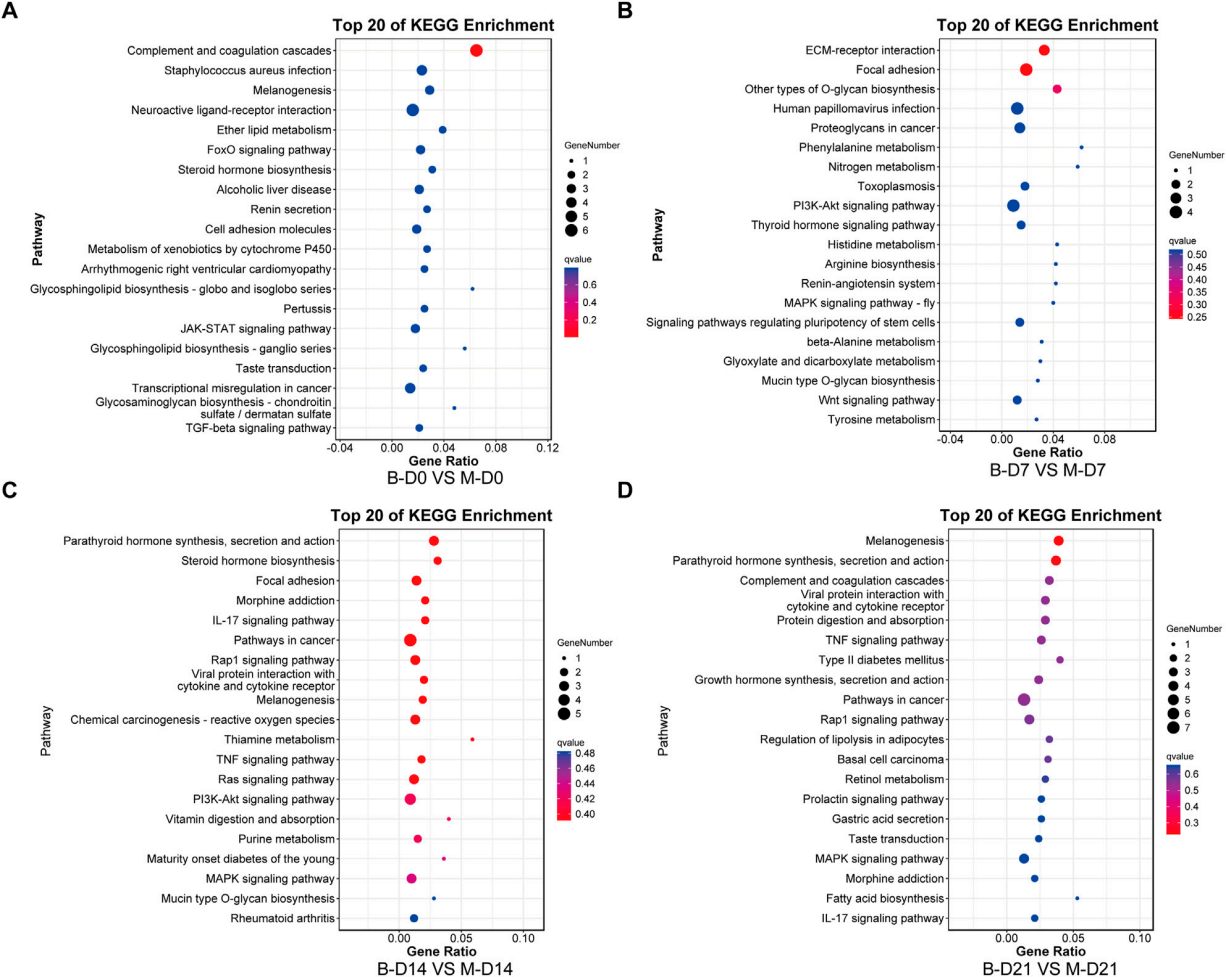
KEGG enrichment analysis of DEGs between MSMSCs and PMSCs. **(A)** Before osteogenic induction. **(B)** At 7 days after osteogenic induction. **(C)** At 14 days after osteogenic induction. **(D)** At 21 days after osteogenic induction. The top 20 most significantly enriched KEGG pathways at each time point were identified based on FDR <0.05. The horizontal axis represents the enrichment factor, and the vertical axis represents the enriched pathway. Red indicates a smaller FDR (more significant enrichment), and blue indicates a larger FDR (less significant enrichment). Legend: “B-D0 vs. M-D0” = “PMSCs at day 0” versus “MSMSCs at day 0”; “B-D7 vs. M-D7” = “PMSCs at day 7” versus “MSMSCs at day 7”; “B-D14 vs. M-D14” = “PMSCs at day 14” versus “MSMSCs at day 14”; “B-D21 vs. M-D21” = “PMSCs at day 21” versus “MSMSCs at day 21”; DEGs = differentially expressed genes; KEGG = Kyoto Encyclopedia of Genes and Genomes.

The top 10 most significantly enriched GO pathways at each time point were identified across the three canonical GO functional categories—molecular function (MF), cellular component (CC), and biological process (BP)—based on fold enrichment >1.5 and FDR <0.05. Combined with KEGG enrichment results, MSMSCs and PMSCs exhibit distinct temporal dynamic evolution patterns during osteogenic induction, as shown in Figures 5, 6.

At the initial induction stage (D0), DEGs predominantly mediate basic cellular functions, encompassing signal molecule binding, maintenance of cell periphery structures, and anatomical structure development. The FoxO signaling pathway serves as a core regulatory hub during this baseline phase, governing fundamental cellular metabolism and stress responses (Figures 5A, 6A).

At 7 days after osteogenic induction, the DEGs between MSMSCs and PMSCs were predominantly associated with biological processes including response to stimulus and cell proliferation, cellular components such as the extracellular matrix (ECM), and molecular functions like signaling molecule-receptor binding. Concurrently, KEGG pathway analysis revealed a significant enrichment of DEGs in the PI3K-Akt signaling pathway, underscoring its critical role in mediating early-stage osteogenic responses related to cell adhesion, proliferation, and signal transduction (Figures 5B, 6B).

From day 7 to day 14 of induction, MSMSCs and PMSCs enter a critical phase of osteogenic signal activation. Molecular functions continue to focus on receptor binding and ligand-receptor interaction; meanwhile, cellular components gradually shift toward the extracellular matrix; biological processes are dominated by stimulus responses. The PI3K-Akt signaling pathway remains activated, and KEGG enrichment revealed the involvement of the IL-17 signaling pathway on day 14, further accelerating osteogenic differentiation (Figures 5C, 6C).

By day 21, MSMSCs and PMSCs progress to the matrix mineralization stage, characterized by significantly enhanced molecular structure activity, maturation of extracellular matrix functions, and the TNF signaling pathway emerging as the core regulatory pathway (Figures 5D, 6D). The shift from basal cell function (D0) to osteogenic signaling (D7-D14) and matrix mineralization (D21) in both cell types highlights the temporal coordination of pathways critical for bone formation.

In summary, the molecular differences between MSMSCs and PMSCs at various stages of osteogenic induction largely reflect the dynamic evolution of signaling pathways such as adhesion-proliferation signaling (early stage), osteogenic signaling pathways (mid-stage), and matrix mineralization (late stage). This finding systematically reveals key molecular events at each stage of osteogenic differentiation from multiple aspects (molecular function, cellular component, and signaling regulation), providing a critical theoretical basis for dissecting the mechanistic differences in osteogenic differentiation between the 2 cell types and exploring applications in bone tissue engineering.

### 3.6 Gene analysis of biological processes related to osteogenic differentiation of MSMSCs

**FIGURE 7:**
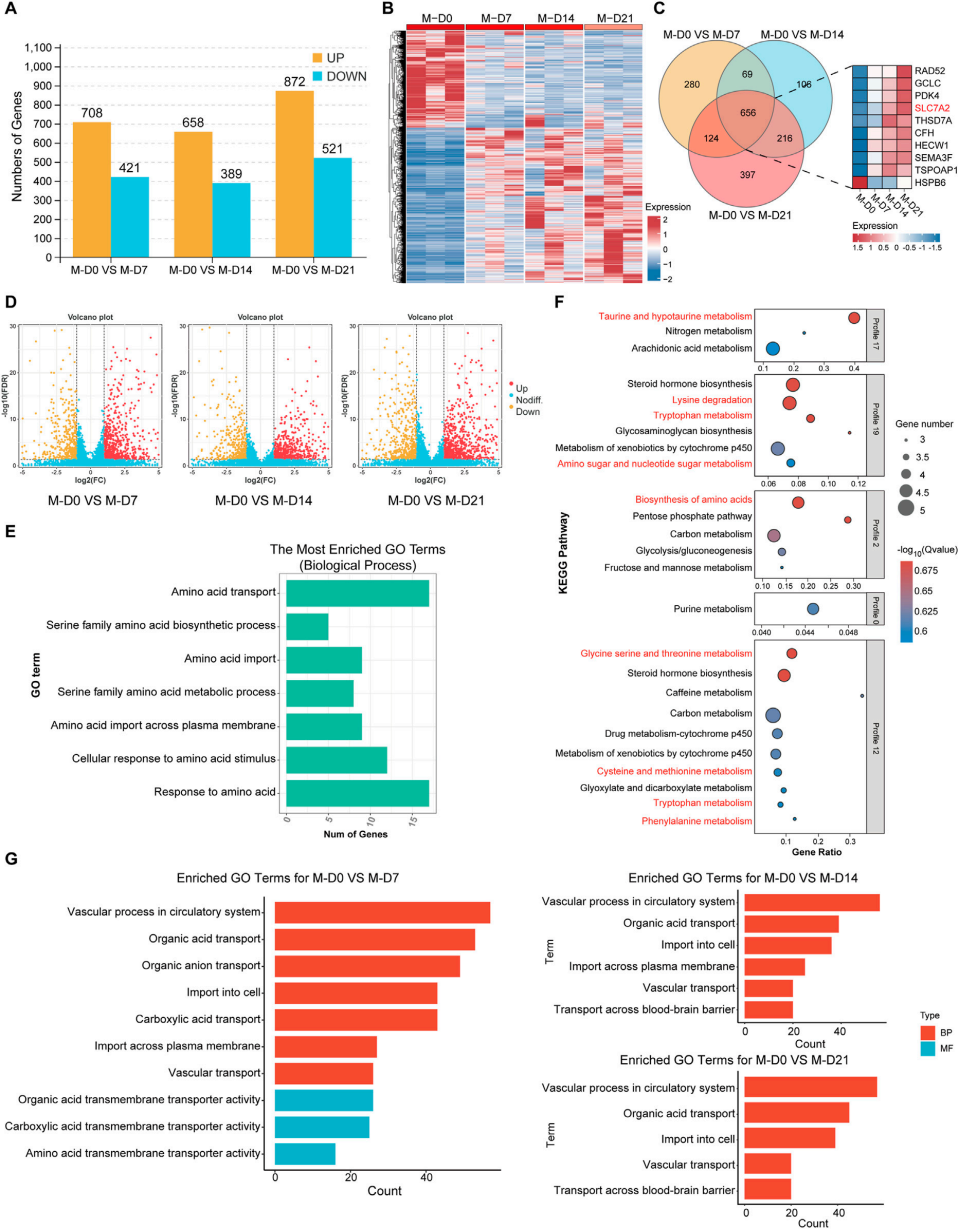
Gene analysis of biological processes related to osteogenic differentiation of MSMSCs. **(A)** Numbers of DEGs during osteogenic differentiation of MSMSCs at different time points. Yellow represents upregulated genes, and blue represents downregulated genes. **(B)** Heat map showing the clustering of gene expression in MSMSCs at different time points. Red represents higher expression, and blue represents lower expression. **(C)** Venn diagram of DEGs identified in M-D0 vs. M-D7, M-D0 vs. M-D14, and M-D0 vs. M-D21 comparisons. The number in each overlapping region represents the count of common DEGs (co-DEGs). Additionally, a bar plot displays the top 10 most significantly differentially expressed genes across these time-course comparisons. **(D)** Volcano plot showing all profiled genes. Red represents upregulated genes, yellow represents downregulated genes,and blue represents non-DEGs, respectively. Statistical analysis detected DEGs between time points (judging criteria: FDR <0.05 and ≥2-fold change in FPKM). **(E)** GO enrichment analysis of amino acid metabolism-related biological processes during MSMSCs’ osteogenic differentiation (horizontal axis: number of genes; vertical axis: GO terms). **(F)** KEGG pathway enrichment analysis of amino acid metabolism-related pathways during MSMSCs’ osteogenic differentiation (horizontal axis: enrichment factor; vertical axis: KEGG pathways). A higher enrichment factor indicates greater pathway enrichment, and a lower FDR value denotes more significant enrichment of DEGs in the pathway. **(G)** GO enrichment analysis of biological processes related to SLC7A2-mediated L-arginine transport (horizontal axis: number of genes; vertical axis: GO terms). Legend: “M-D0” = MSMSCs at day 0; “M-D7” = MSMSCs at day 7; “M-D14” = MSMSCs at day 14; “M-D21” = MSMSCs at day 21; GO = Gene Ontology; DEGs = differentially expressed genes; KEGG = Kyoto Encyclopedia of Genes and Genomes; SLC7A2 = solute carrier family 7 member 2.

DESeq2 software was used, and DEGs were screened based on criteria of FDR <0.05 and |log_2_FC| ≥2.0. The DEGs of MSMSCs before osteogenic differentiation and at 7, 14, and 21 days after differentiation were screened out, and a histogram and cluster heat maps were drawn (Figures 7A,B). DEGs at different time points during MSMSCs osteogenic induction were analyzed, and a Venn diagram was drawn. There were 656 common DEGs at 7, 14, and 21 days. The top 10 genes with the highest differential expression significance included nine upregulated genes: RAD52, GCLC, PDK4, SLC7A2, THSD7A, CFH, HECW1, SEMA3F, and TSPOAP1, and one downregulated gene: HSPB6 (Figure 7C). A volcano map was drawn to visually show the DEGs between groups, and each point in the map represents a gene (Figure 7D).

The biological processes related to amino acid metabolism, such as response to amino acids, amino acid transport, and cell response to amino acid stimulation after osteogenesis induction of MSMSCs were analyzed. The genes associated with signaling pathways included SLC7A2, SLC7A5, SLC1A5, SLC6A6, SLC7A11, GCLC, and COL16A1, etc., (Figure 7E). KEGG signaling pathways related to amino acid metabolism in different MSMSCs osteogenic differentiation were analyzed, and related signaling pathways included amino acid biosynthesis, lysine degradation, taurine and hypotaurine metabolism, and tryptophan metabolism pathway (Figure 7F).

A Gene Ontology (GO) enrichment analysis was performed on the DEGs obtained from the comparisons of M0 vs. M7, M14, and M21 respectively. Pathways associated with SLC7A2-mediated L-arginine transport were selected and presented. These results indicate that SLC7A2 primarily influences the osteogenic differentiation process, particularly the osteogenic-related endocytosis process (Figure 7G), by regulating the transport of metabolites across the cell membrane, including amino acids, carboxylic acids, and organic acids.

In addition, GO pathway enrichment analyses revealed that during days 7, 14, and 21 of osteogenic differentiation, multiple metabolic pathways—including carbohydrate, lipid, and amino acid metabolism—were significantly altered. Notably, from day 14 to day 21, pathways related to NO synthesis and mTOR signaling also began to exhibit changes (Details can be found in the supplementary materials S2).

Among these, SLC7A2 was identified as one of the top 10 common DEGs in MSMSCs across these time points (before osteogenic differentiation and at days 7, 14, and 21 post-differentiation), with a >4-fold upregulation at day 7 and FDR <0.01. Furthermore, SLC7A2 was significantly enriched in the “amino acid metabolism” pathway. As a member of the SLC7 family, SLC7A2 is known to mediate the transmembrane transport of L-arginine and other basic amino acids, and prior studies have demonstrated that L-arginine metabolism acts as a critical regulator of osteogenesis. Therefore, we proposed SLC7A2 as a key gene in amino acid metabolism during MSMSCs’ osteogenic differentiation.

### 3.7 Expression of SLC7A2 and construction of lentivirus during MSMSCs osteogenesis

**FIGURE 8:**
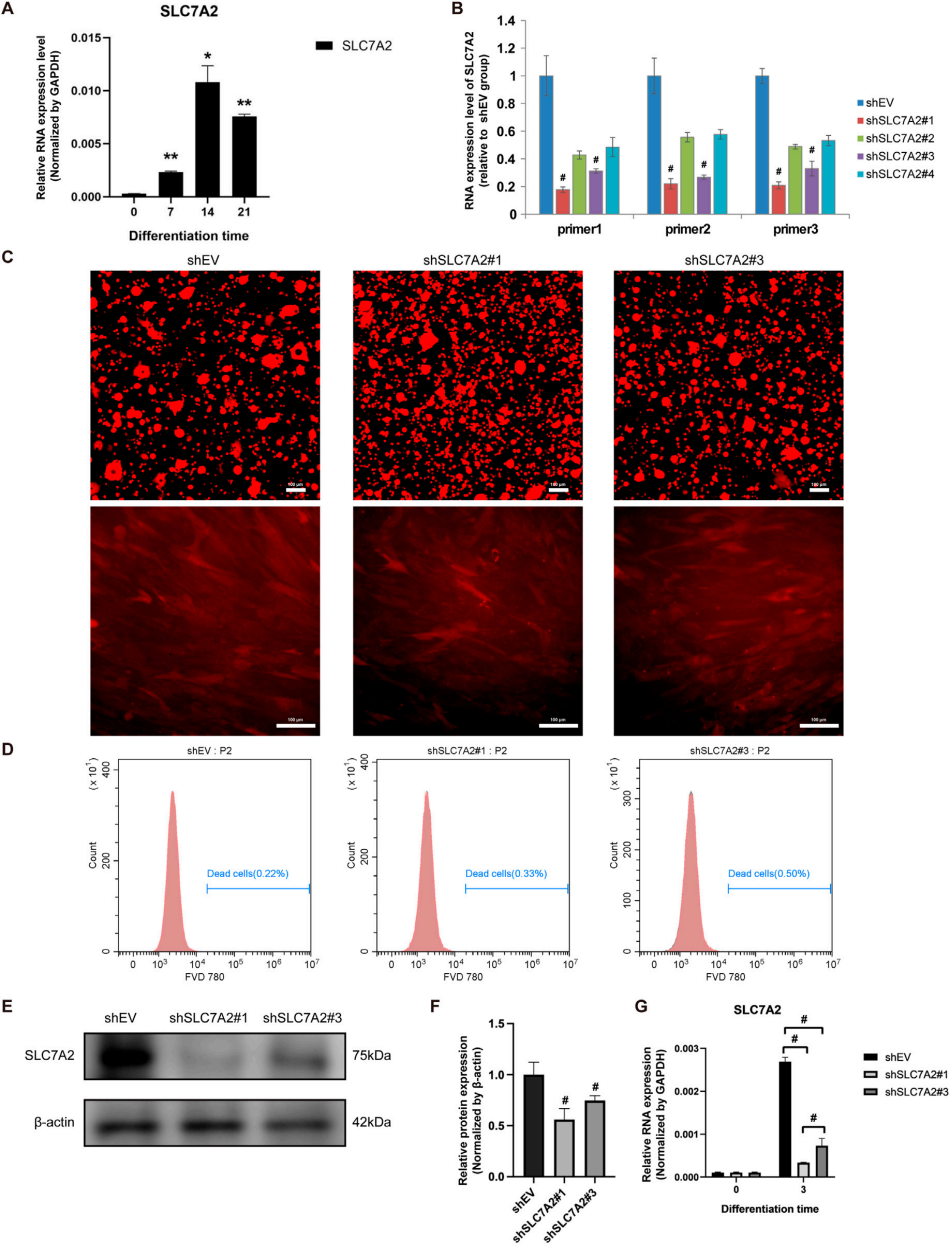
Expression of SLC7A2 and construction of lentivirus during osteogenesis of MSMSCs. **(A)** qPCR showed that SLC7A2 mRNA levels were significantly upregulated during osteogenesis, reaching a peak on day 14. **(B)** Four lentiviral shRNA vectors targeting SLC7A2 were constructed. qPCR showed that SLC7A2 mRNA levels in MSMSCs transfected with these vectors were decreased to different degrees compared with the shEV control group. **(C)** Lentivirus packaging and transfection: fluorescence microscopy images of MSMSCs 72 h after shSLC7A2 lentivirus transfection. Scale bars = 100 µm. **(D)** FVD780/Calcein staining for flow cytometry analysis of cell viability 72 h post-lentivirus transfection: flow cytometry dot plots depicting the ratio of dead cells. **(E)** WB analysis of SLC7A2 protein expression in MSMSCs 72 h after shSLC7A2 transfection. **(F)** Bar chart showing SLC7A2 protein expression (normalized to β-actin) quantified from WB results. **(G)** qPCR analysis of SLC7A2 mRNA levels in MSMSCs 72 h after shSLC7A2 transfection. Target mRNA levels were normalized to GAPDH. Results are representative of three independent experiments. Error bars indicate the standard error of the mean. * and ** indicate *P* < 0.05 and *P* < 0.01, respectively, when comparing MSMSCs at D7, D14, and D21 with D0 within the same group. # indicates *P* < 0.05 when comparing the shSLC7A2 and shEV groups. *Legend*: shEV = shRNA empty vector; shSLC7A2 = shRNA targeting solute carrier family 7 member 2; GAPDH = Glyceraldehyde 3-phosphate dehydrogenase; qPCR = quantitative PCR; WB = Western blotting.

At 7, 14 and 21 days after MSMSCs osteogenesis induction, the qPCR results showed that the mRNA level of SLC7A2 was significantly upregulated, reaching a peak on day 14 and then exhibiting a slight decrease on day 21 (*P* < 0.05) (Figure 8A).

Four lentivirus vectors were constructed, shSLC7A2#1, shSLC7A2#2, shSLC7A2#3, and shSLC7A2#4, and a control group shEV. The results of qPCR showed that the SLC7A2 mRNA of the four lentiviral vectors was decreased in different degrees compared with the control group. Among them, the SLC7A2 mRNA levels in the shSLC7A2#1 and shSLC7A2#3 groups were significantly decreased (*P* < 0.05) (Figure 8B).

MSMSCs were then divided into three groups, control group (shEV), shSLC7A2#1 group, and shSLC7A2#3 group. After lentivirus packaging and transfection of MSMSCs, cell morphology was observed under an inverted microscope (Leica, Germany) for mCherry fluorescence imaging. At 72 h post-transfection, cells in all three groups exhibited healthy growth and uniform distribution (Figure 8C). Cells were harvested and analyzed by flow cytometry (BD, United States) to quantify live (Calcein^+^/FVD780^-^) and dead (Calcein^−^/FVD780^+^) populations. All three groups showed ≥95% viability, confirming high cell viability (Figure 8D). Western blotting and bar charts normalized to GAPDH showed that the expression of SLC7A2 protein in the shSLC7A2#1 and shSLC7A2#3 groups was lower than in the shEV group (Figures 8E,F). Total RNA was extracted from the cells of the three groups, and the expression of SLC7A2 mRNA was detected by qPCR. The results showed that 72 h after transfection of MSMSCs with shSLC7A2, the levels of SLC7A2 mRNA in the shSLC7A2#1 and shSLC7A2#3 groups were significantly lower than those in the control group (*P* < 0.05) (Figure 8G). These results confirmed successful lentiviral construction and efficient SLC7A2 knockdown in MSMSCs, which could significantly downregulate the expression of SLC7A2 and the transfection efficiency reached the experimental requirements.

### 3.8 Osteogenic differentiation staining, osteogenic-related genes and protein expression of MSMSCs transfected with shSLC7A2

**FIGURE 9:**
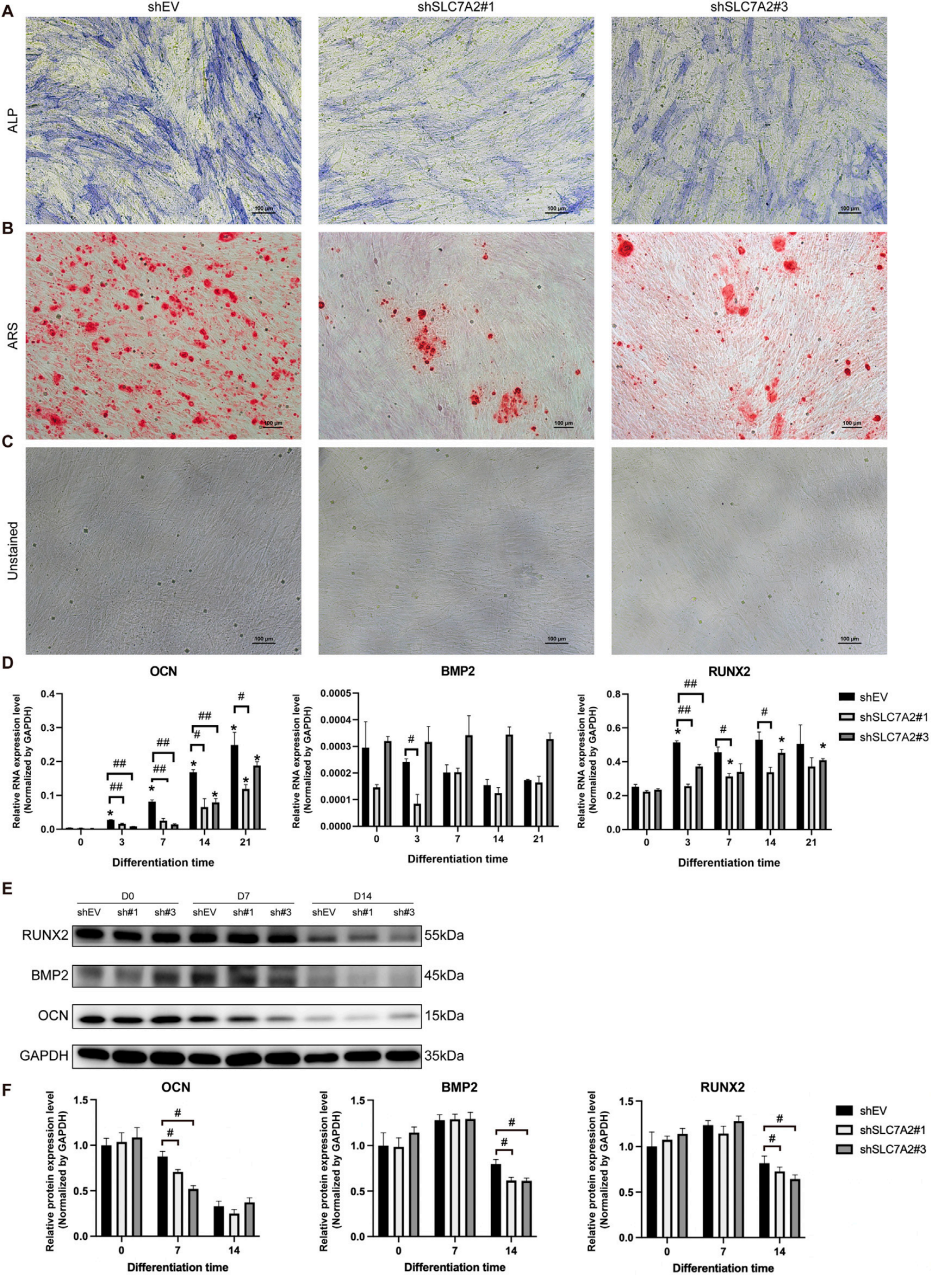
Osteogenic differentiation staining, osteogenic-related genes, and protein expression of MSMSCs transfected with shSLC7A2. **(A)** ALP staining. **(B)** ARS staining. **(C)** Unstained MSMSCs transfected with shSLC7A2 at 21 days after osteogenic induction. Scale bars = 100 µm. **(D)** qPCR analysis of OCN, RUNX2, and BMP2 mRNA expression on D3, D7, D14, and D21 after osteogenic induction in shSLC7A2-transfected MSMSCs. Target mRNA levels were normalized to GAPDH. **(E)** WB analysis of OCN, RUNX2, and BMP2 protein expression at t D0, D7 and D14 after osteogenic induction in shSLC7A2-transfected MSMSCs. **(F)** Bar chart showing OCN, RUNX2, and BMP2 protein levels (normalized to GAPDH) quantified from WB analysis in shSLC7A2-transfected MSMSCs at D0, D7, and D14 after osteogenic induction. Results are representative of three independent experiments. Error bars indicate the standard error of the mean. * indicates *P* < 0.05 when comparing shSLC7A2-transfected MSMSCs at D3, D7, D14, and D21 with D0 within the same group. # *P* < 0.05 and ## *P* < 0.01 indicate statistically significant differences at the 0.05 and 0.01 levels, respectively,between the shSLC7A2 and shEV group at the same time point. L*egend*: shEV = short hairpin RNA empty vector; shSLC7A2 = Short hairpin RNA targeting solute carrier family 7 member 2; GAPDH = Glyceraldehyde 3-phosphate dehydrogenase; qPCR = quantitative PCR; WB = Western blotting; ALP: alkaline phosphatase; ARS:Alizarin Red S staining. OCN: Osteocalcin; RUNX2:Runt-related transcription factor2; BMP2: Bone Morphogenetic Protein 2.

MSMSCs were transfected with shEV, shSLC7A2#1, and shSLC7A2#3, respectively. At day 21 after osteogenic induction, ALP and ARS staining were performed at Day 21 post-induction. Positive calcium staining was observed in all groups, but the staining intensity and number of calcium nodules were significantly lower in shSLC7A2#1 and shSLC7A2#3 groups compared to the control group (Figures 9A–C).

Total RNA was extracted before induction, and at 3, 7, 14, and 21 days after induction. The results of qPCR showed that in the shSLC7A2#1 group, OCN mRNA expression increased gradually from Day 3–21 post-induction but remained significantly downregulated compared to the control group at all time points (*P* < 0.05). 3 days after osteogenic induction, the mRNA expression of BMP2 in the shSLC7A2#1 group was decreased compared with the control group (*P* < 0.05). At day 7, and 14 after osteogenic induction, the mRNA expression of RUNX2 was increased in different degrees compared with that before induction, and was significantly lower than that of the control group (*P* < 0.05) (Figure 9D).

In the shSLC7A2#3 group, the mRNA level of OCN increased gradually with time after osteogenic induction, and was lower than that of the control group at day 3, 7, and 14 (*P* < 0.05). Compared with the control group, the mRNA expression of BMP2 was higher in shSLC7A2#3 group. In shSLC7A2#3 group, the mRNA expression of RUNX2 was increased in different degrees after osteogenic induction compared with that before induction, and decreased compared with the control group. The difference was statistically significant at day 3 (*P* < 0.05) (Figure 9D).

Total protein was extracted before osteogenic induction, and on day 7 and day 14 after induction in the three groups, followed by Western blotting. The results showed that, at day 7 after osteogenic induction, the expression levels of OCN in the shSLC7A2#1 and shSLC7A2#3 groups were lower than those in the control group. Moreover, at day 14 after osteogenic induction, the protein levels of BMP2 and RUNX2 in the shSLC7A2#1 and shSLC7A2#3 groups were also lower than those in the control group (Figures 9E,F).

These results show that the osteogenic ability of the cells was decreased after transfecting MSMSCs with shSLC7A2, and lower than that of the control group.

## 4 Discussion

The formation of new bone in the maxillary sinus floor is crucial to ensure initial stability after dental implantation and is also the foundation for successful dental implantation. One of the factors that determines the formation of new bone in this area is the osteogenic differentiation potential of MSMSCs.

Although both MSMSCs and PMSCs exhibit osteogenic potential, the intensity and speed of osteogenesis differ between the two cell types at various time points post-induction. Therefore, investigating the gene expression profiles and transcriptional regulatory mechanisms of osteogenic differentiation in MSMSCs and PMSCs is critical to elucidate their differentiation potential and gene expression patterns.

RNA was collected from a total of 24 samples of MSMSCs and PMSCs at different time points before and after osteogenic differentiation using RNA-seq technology, and enrichment analysis of DEGs was performed. The results showed that both MSMSCs and PMSCs had characteristics of stem cells, and exhibited similar CD marks expression patterns before osteogenic induction, with high expression of CD105, CD73, and CD90. Meanwhile, their expression of CD73 and CD90 was higher than that of fibroblasts, and CD34 and CD45 levels were lower than those in fibroblasts, which is consistent with that reported by Onizuka et al. (2020). In their study, MSCs were isolated, cultured, and identified from three different bones including ilium, maxilla and mandible, and RNA sequencing was performed. The results showed that the expression patterns of CD marks in the three different types of MSCs were similar. Furthermore,the expression of CD105, CD73, and CD90 were high while the expression of CD45 was almost absent. All of these results are consistent with the standard of MSCs identification defined by the International Society for Cellular Therapy (ISCT) (Choudhery et al., 2022). The results also further validated our previous findings that MSMSCs and PMSCs not only morphologically conformed to the characteristics of MSCs, but also *in vitro* cultured both had osteogenic differentiation potential and showed similar expression patterns, and the CD marks expression profiles were consistent with the ISCT criteria for MSCs.

MSMSCs and PMSCs exhibited partially similar gene expressions during early (7 days) and middle (14 days) osteogenic induction, primarily associated with stimulus response and signal receptor binding pathways. On the other hand, however, differences in transcriptional regulation were observed. GO and KEGG enrichment analysis showed that MSMSCs and PMSCs have different biological processes in which specific, highly-expressed genes are involved. The specific biological processes of PMSCs include cell proliferation, extracellular matrix, signal receptor binding, and PI3K-Akt, Wnt, and IL-17 signaling pathways, etc. The ability of PMSCs to sense and perceive external stimuli, especially chemical stimuli and changes in extracellular matrix, and transmit the stimuli to produce a series of reactions appears to be stronger than that of MSMSCs, which is consistent with the results of a previous study (Li et al., 2019). The slow response of MSMSCs to external stimuli may also be a reason for their long-term stability. At the late stage of osteogenic induction (21 days), the DEGs between MSMSCs and PMSCs were mainly associated with anatomical structure development and bone mineralization, suggesting that the differences between the two groups decreased gradually with time. It has been shown that bone MSCs in different parts of the maxillofacial region are all derived from neural crest cells, and the expression pattern identified with RNA-seq is not the same as that of iliac bone derived from mesoderm (Naung et al., 2019). Although both MSMSCs and PMSCs are MSCs derived from maxillofacial bones and have the same CD marks expression characteristics, their *in vivo* microenvironments are distinct, which may lead to subtle differences in their *in vivo* differentiation potentials despite overall similarity.

We further analyzed GO and KEGG pathway enrichment of DEGs at different time points during MSMSCs osteogenic processes. This analysis indicates that MSMSCs are closely associated with amino acid metabolism during osteogenic processes, which is consistent with the results of prior studies (Nakaya et al., 2014; Chiu et al., 2021). Both MSMSCs osteogenic differentiation and matrix production require biosynthesis, which greatly increases the metabolic demand of osteoblasts (Nicklin et al., 2009). Amino acid metabolism is critical for osteoblast development and function, particularly in maintaining intracellular homeostasis. The proliferation of osteoblasts requires increased cellular energy and acquisition of amino acids to produce nutrients needed for cell division (Nakashima et al., 2002). The consumption of amino acids is transcriptionally regulated and is rapidly upregulated during differentiation in response to osteoinductive signals. More importantly, mutations in amino acid transport and uptake-related proteins resulted in a significant decrease in the proliferation capacity of osteoblasts and a significant reduction in bone formation (Ornitz and Marie, 2002). Similarly, the proliferation, differentiation, and maturation of MSMSCs also requires a continuous supply of a large number of amino acids to maintain proliferation and bone matrix production.

Our results showed that the expressions of amino acid metabolism-related genes in the SLC7 family, especially SLC7A2, were upregulated in the osteogenesis of MSMSCs. We speculate that the SLC7A2 gene may be a key gene that plays a regulatory role in the osteogenesis of MSMSCs. Further *in vitro* experiments showed that SLC7A2 knockdown significantly reduced the mRNA expression of OCN and RUNX2 in MSMSCs compared with the control group (*P* < 0.05). In our study, This molecular change was consistent with impaired osteogenic function, as evidenced by decreased matrix mineralization in ARS staining and ALP activity. It has been shown that RUNX2 is an early transcription factor that regulates the expression of osteoblast-specific genes and promotes osteogenic differentiation of stem cells by regulating multiple osteogenesis-related genes (Narayanan et al., 2019; Vimalraj et al., 2015). OCN is an indispensable specific transcription factor in the metabolism of bone tissue, and its level can directly reflect the calcification degree of bone (Ge et al., 2019). It is also recognized as a marker of late osteogenesis mediated by RUNX2 (Wu et al., 2019). BMP2 is a growth factor known to induce osteoblast differentiation and osteogenic gene expression, thus playing an important role in bone repair (Zhou et al., 2016; Wang et al., 2018; Florian et al., 2019; Zhang et al., 2022; Singh and Ecker, 2018).

It has been reported that SLC7A5 and SLC7A11 are both expressed in osteoblasts, which mediate the transport of essential amino acids into or out of osteoblasts, thus affecting the proliferation and differentiation of osteoblasts (Kandasamy et al., 2018; Ni et al., 2021). Overexpression of SLC7A11 in osteoblasts can positively regulate the generation of glutathione (GSH) at the molecular level, and negatively regulate the expression of RUNX2 at the transcription level, thus affecting the process of osteogenesis. SLC7A5 promotes the proliferation and differentiation of osteoblasts, and maintains the balance of bone metabolism through mTOR (Ni et al., 2021). The L-arginine transporter SLC7A2 provides key substrates for the growth of cells and organelles by regulating the transport of amino acids, thus affecting cell proliferation and differentiation. L-arginine is a nitric oxide donor, which can improve the hypoxia tolerance in tissues and cells (Polewski et al., 2017). As a ubiquitous biological messenger and immunomodulatory molecule, NO can rapidly transmigrate through the cell membrane to act on the surrounding cells, and plays an important regulatory role in the process of bone formation and bone resorption (Sun et al., 2020). However,the specific mechanisms by which SLC7A2—acting as an L-arginine transporter—regulates downstream cellular processes remain unclear.

SLC7A2 functions as a critical gateway for L-arginine entry into MSMSCs, and its transport activity directly feeds into two core metabolic pathways that drive osteoblast differentiation. First, Chevalley et al. have demonstrated that arginine (Arg) may stimulate bone formation directly or indirectly by enhancing local IGF-1 production (Chevalley et al., 1998). Second, L-arginine serves as the rate-limiting substrate for nitric oxide synthases (NOS), which generate NO—a signaling molecule proven to enhance bone formation by activating the cyclic guanosine monophosphate (cGMP)-Protein Kinase G (PKG) pathway and upregulating RUNX2, the master transcription factor of osteogenesis (Kalyanaraman et al., 2018). Moreover, L-arginine is a precursor for polyamine biosynthesis via the enzyme ornithine decarboxylase (ODC), and polyamines have recently been identified as central regulators of MSCs lineage commitment. As shown by Tabbaa et al., polyamine pathway enzymes are differentially regulated in models of altered mechanical loading, and mTORC1 activation may regulate the polyamine metabolic pathway in skeletal muscle (Tabbaa et al., 2021). Furthermore, some findings suggest that L-arginine enhances mitochondrial functions by upregulating gene expression through the mTOR signaling pathway (Zhou et al., 2024). In conclusion, these data position SLC7A2 as a pivotal node that may connect L-arginine metabolism to both the NO-cGMP and polyamine-mTORC1 axes—two established pathways that coordinate osteogenic differentiation in MSCs.

In gene function research, commonly employed technologies include Clustered Regularly Interspaced Short Palindromic Repeats/CRISPR-associated protein 9(CRISPR/Cas9), small interfering RNA (siRNA), and shRNA vectors. CRISPR/Cas9 functions at the DNA level, leveraging guide RNA (gRNA) to direct the Cas9 nuclease for site-specific DNA cleavage, thereby facilitating precise genome editing, including gene knockout, knock-in, or base substitution. While this system enables permanent genomic modifications, it necessitates the design and construction of gRNA expression vectors, requiring sophisticated experimental methodologies and meticulous handling. When applied *in vivo*, potential hurdles associated with vector delivery efficiency and safety must be carefully addressed. In contrast, siRNA exhibits an inherently short half-life in cells and is susceptible to nuclease degradation, typically achieving only transient gene silencing. Continuous transfection is thus required to maintain the silencing effect. In comparison to these methods, lentiviral-delivered shSLC7A2 exhibits several distinct advantages. Notably, it can be efficiently transduced into diverse cell types, including primary cells, stem cells, and *in vivo* models that are often refractory to transfection. Additionally, it mediates stable and sustained gene silencing, ensuring consistent suppression of SLC7A2 expression over an extended period. Given the limitations of CRISPR/Cas9 and siRNA, we chose lentiviral-mediated transfection of shSLC7A2 to ensure stable and prolonged suppression of SLC7A2 expression in this investigation.

By systematically comparing the transcriptional and molecular differences between MSMSCs and PMSCs, this study offers novel insights and identifies that SLC7A2 is required for the efficient osteogenic differentiation of MSMSCs. However, several limitations warrant further investigation. First, this study lacks rescue experiments to confirm the sufficiency of SLC7A2 in promoting osteogenesis. Additionally, we did not perform complementary experiments such as SLC7A2 overexpression in MSMSCs or L-arginine supplementation to reverse the osteogenic defects in SLC7A2-silenced MSMSCs.More critically, while our shRNA-mediated knockdown data demonstrate that SLC7A2 is necessary for the efficient osteogenic differentiation of MSMSCs, the specific mechanisms by which SLC7A2—acting as an L-arginine transporter—regulates downstream osteogenic cellular processes remain unclear. Future research should involve overexpressing SLC7A2 in MSMSCs to test whether increased SLC7A2 levels are sufficient to upregulate the mRNA expression of key osteogenic markers (RUNX2, OCN, BMP2) and enhance matrix mineralization. Future studies should also supplement SLC7A2-silenced MSMSCs with L-arginine to determine if restoring L-arginine metabolism can reverse the observed osteogenic defects. These experiments will help validate the causal role of SLC7A2-mediated L-arginine transport in MSMSCs osteogenesis and provide more definitive insights into its therapeutic potential for craniofacial bone regeneration.

In conclusion, comparative RNA-Seq of MSMSCs and PMSCs revealed that their transcriptional profiles are consistent with MSCs characteristics and exhibit highly similar gene expression patterns, while key transcriptional disparities were identified. Notably, our findings further highlight that the osteogenic differentiation of MSMSCs is intricately linked to amino acid metabolism and the SLC7 family. The L-arginine transporter SLC7A2 may be required for the efficient osteogenic differentiation of MSMSCs. These findings provide a potential theoretical basis for improving the osteogenic differentiation capacity of MSMSCs and serve as a reference for exploring novel strategies to promote site-specific osteogenesis following maxillary sinus elevation.

## Data Availability

The datasets generated and analysed during the current study are available in the Genome Sequence Archive for Human (GSA for Human) repository, and the accession number to dataset is HRA007100.
